# De novo biosynthesis of τ-cadinol in engineered *Escherichia coli*

**DOI:** 10.1186/s40643-022-00521-7

**Published:** 2022-03-21

**Authors:** Yue Sun, Shaoting Wu, Xiao Fu, Chongde Lai, Daoyi Guo

**Affiliations:** 1 College of Bioscience and Bioengineering, Jiangxi Agricultural University, Nanchang, 330045 China; 2 Key Laboratory of Organo-Pharmaceutical Chemistry, Gannan Normal University, Ganzhou, 341000 Jiangxi Province China

**Keywords:** Metabolic engineering, *Escherichia coli*, τ-Cadinol, MVA

## Abstract

τ-Cadinol is a sesquiterpene that is widely used in perfume, fine chemicals and medicines industry. In this study, we established a biosynthetic pathway for the first time in engineered *Escherichia coli* for production of τ-cadinol from simple carbon sources. Subsequently, we further improved the τ-cadinol production to 35.9 ± 4.3 mg/L by optimizing biosynthetic pathway and overproduction of rate-limiting enzyme IdI. Finally, the titer was increased to 133.5 ± 11.2 mg/L with a two-phase organic overlay-culture medium system. This study shows an efficient method for the biosynthesis of τ-cadinol in *E. coli* with the heterologous hybrid MVA pathway.

**Graphical Abstract:**

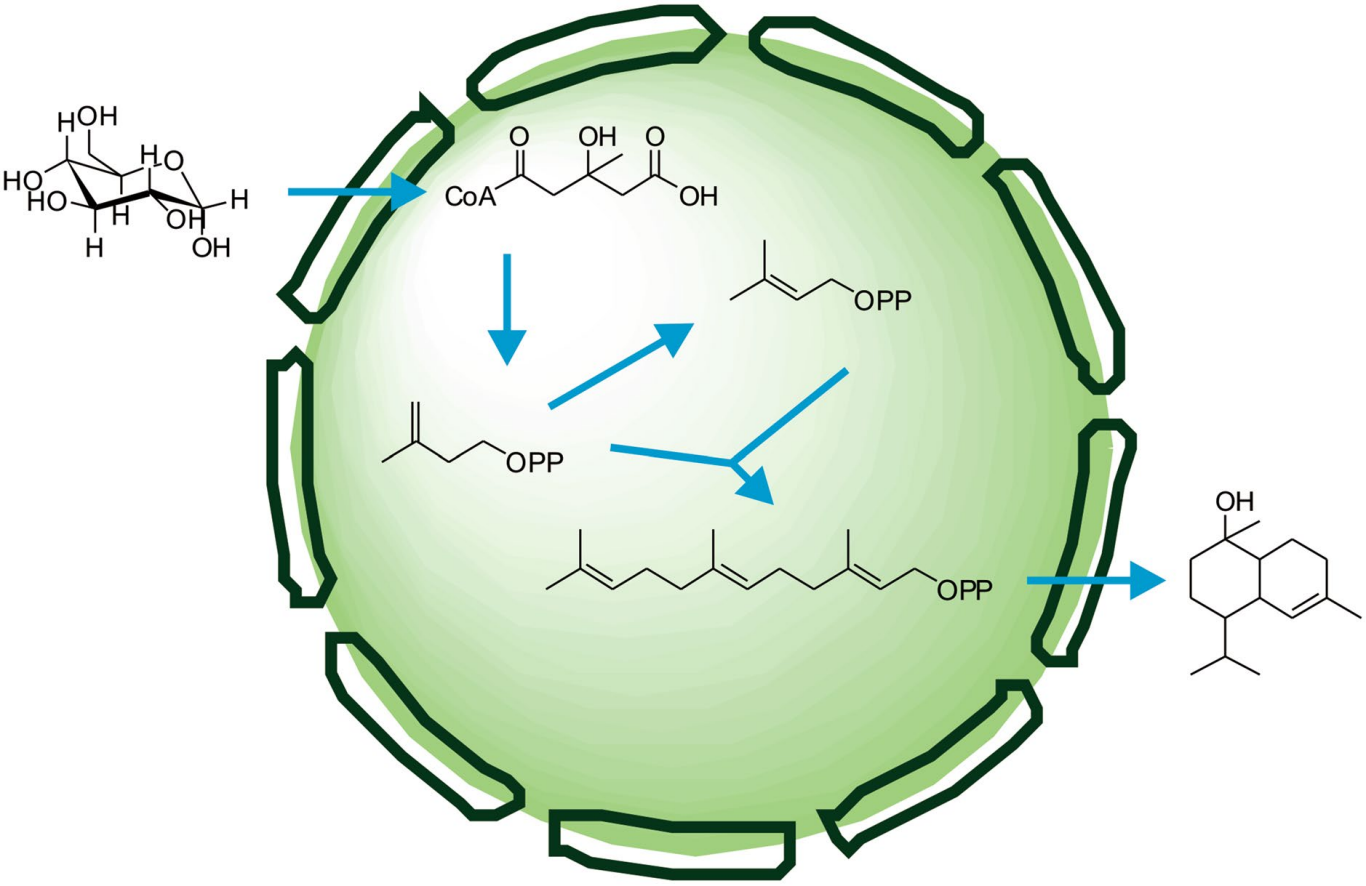

**Supplementary Information:**

The online version contains supplementary material available at 10.1186/s40643-022-00521-7.

## Introduction

Terpenoids are the largest class of secondary metabolites found in nature. So far, more than 50,000 terpenoids have been identified. Due to the diversity of biological functions, many terpenoids are widely used in industries such as chemistry, perfume, medicine and nutraceuticals (Li et al. 2020). τ-Cadinol is a sesquiterpene widely distributed in plants, insects, and microorganisms with a wide range of application prospects (Pascal et al. 2003; Yamada et al. 2015). The usual way to get it mainly comes from the heartwood of *Cryptomeria japonica* and Myrrh (Andersson et al, 1997; Narita et al. 2006). τ-Cadinol is widely used in essential oils and spices because of its special fragrant odor, which makes it a highly sought-after fragrances and scent compounds in the food and cosmetics industries. τ-Cadinol also displays extensive biological activity. As a pharmacological component of Myrrh in Somali traditional medicine, it is used for the treatment of diarrhea and wounds (Claeson et al. 1991a, b). It has been reported that τ-cadinol as a calcium antagonist can relax the smooth muscle in the rat aorta, which makes it clinically potentially useful for the treatment of gastric spasms and vasospasm diseases (Claeson et al. 1991a, b). Masao Takei et al. reported the effect of τ-cadinol on dendritic cells (immune response center), and the results showed that τ-cadinol enhanced the differentiation and functional maturation of dendritic cells, which indicates that τ-cadinol can be used for dendritic cell-based cancer immunotherapy (Takei et al. 2006). In addition, it has been reported that τ-cadinol can effectively interact with the cell envelope of *Staphylococcus aureus*, resulting in bacterial lysis and thus bactericidal effect (Claeson et al. 1992). Because of its effective fungicidal and insecticidal action it could be used as a wood preservative in the future (Wu et al. 2005).

**Fig. 1:**
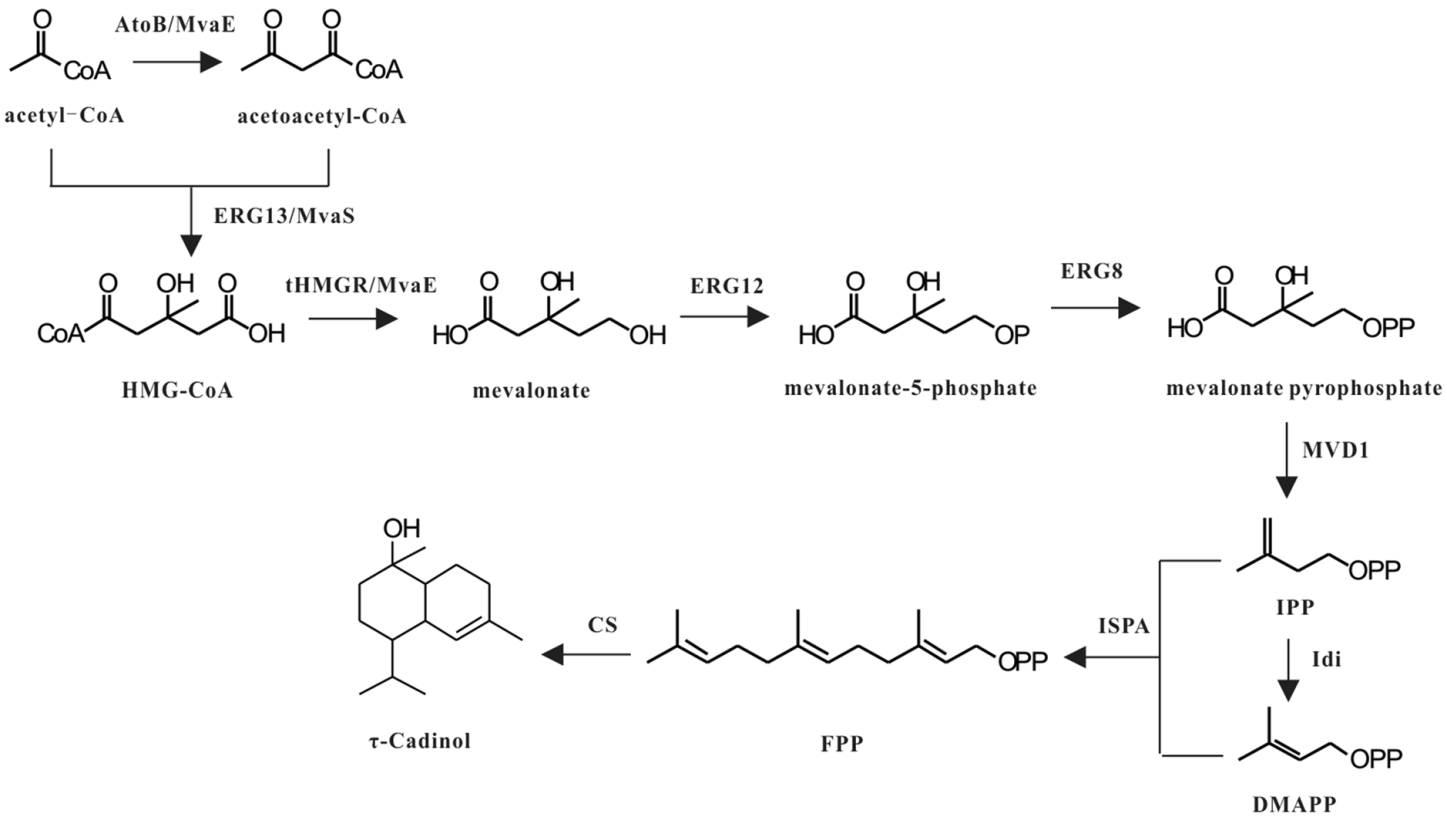
Engineered pathway for the production of τ-cadinol from glucose

Currently, two τ-cadinol synthases from *Lavandula angustifolia* and maize have been identified (Fei et al. 2016; Jullien et al. 2014). There are two pathways used for terpenoid biosynthesis in plants: the mevalonate (MVA) pathway in the cytosol and the methylerythritol phosphate (MEP) pathway in the plastids. Therefore, MVA and MEP pathways may both contribute to τ-cadinol synthesis in plants through metabolite exchanges between cytosol and plastid. The first τ-cadinol synthase, LaCADS, was identified recently in *L. angustifolia*, which produced τ-cadinol as the predominant product and r-cadinene as minor product (Jullien et al. 2014). Subsequently, a terpene synthase ZmTPS7 from maize was characterized as a τ-cadinol synthase (Fei et al. 2016). ZmTPS7 was co-expressed with farnesyl diphosphate synthase IspA in *E. coli*. GC–MS analysis showed that ZmTPS7 reacted with farnesyl diphosphate to form a blend of sesquiterpenoids. The predominant constituent was identified as τ-cadinol. So far there is no promising way to prepare τ-cadinol by biological production method. Therefore, it is necessary to develop microbial cell factories to synthesize τ-cadinol. In this study, we constructed a biosynthetic pathway for the production of τ-cadinol in *E. coli* with the heterologous hybrid MVA pathway (Fig. 1).

## Methods

### Plasmid and strains

The HMG-CoA synthase MvaS_A110G_ and bifunctional acetyl-CoA acetyltransferase/HMG-CoA reductase MvaE genes from *Enterococcus faecalis* were synthesized by Genscript (Yoona et al. 2009; Wu et al. 2019) and amplified using primers MvaS-XbaI/MvaS-SpeI-BamHI and MvaE-XbaI/MvaE-SpeI-BamHI, and ligated into pET28a (+) via XbaI and BamHI to yield plasmid pSY06 and pSY07. The XbaI–XhoI fragment of MvaE from pSY07 was inserted into SpeI and XhoI sites of pSY06 to give pSY08. The XbaI–XhoI fragment of MvaS_A110G_ and MvaE from pDG08 was inserted into XbaI and XhoI sites of pDG30 to give pSY09.

Farnesyl diphosphate synthase IspA gene from *E. coli* was amplified by PCR using primers IspA-XbaI/IspA-SpeI-BamHI and cloned into pET28a (+) with XbaI/BamHI restriction sites, creating plasmid pSY10. τ-Cadinol synthase CS from *L. angustifolia* was codon optimized and synthesized by Genscript (Jullien et al. 2014). CS was amplified by PCR using primers CS-XbaI/CS-SpeI-BamHI and cloned into pET28a (+) with XbaI/BamHI restriction sites, creating plasmid pSY11. CS was then excised from pSY11 with XbaI/XhoI and ligated into SpeI and XhoI sites of pSY10 to create pSY12. The isopentenyl-diphosphate isomerase IdI gene from *E. coli* was amplified by PCR using primers IdI-XbaI/ IdI-XhoI and ligated into SpeI and XhoI sites of pSY12 to create pSY13. The strains, primers and plasmids used in this study are summarized in Tables 1 and 2.

**Table 1:** Primers used in this study

Primer name	Sequence (5′–3′)
MvaS-XbaI	CTATCTAGAAAGAGGAGATATAATGACCATTGGTATTGATAAAATCAGCT
MvaS-SpeI-BamHI	TACGGATCCACTAGTTTAGTTGCGATAGCTGCGCACG
MvaE-XbaI	GTATCTAGAAAGAGGAGATATAATGAAGACCGTTGTGATTATTGACG
MvaE-SpeI-BamHI	TATGGATCCACTAGTTTACTGTTTACGCAGGTCGTTCAGG
IspA-XbaI	GTATCTAGAAAGAGGAGATATAATGGACTTTCCGCAGCAACTCG
IspA-SpeI-BamHI	TATGGATCCACTAGTTTATTTATTACGCTGGATGATGTAGTCCG
CS-XbaI	GTATCTAGAAAGAGGAGATATAATGGCGACGAGCGCGGT
CS-SpeI-BamHI	TATGGATCCACTAGTTTAAAACACCAGCGGATCTAAAAACA
IdI-XbaI	ATCTCTAGAAAGAAGGAGATATAATGCAAACGGAACACGTCATTTTAT
IdI-XhoI	ACATCTCGAGTTATTTAAGCTGGGTAAATGCAGATAATC

**Table 2:** Plasmids and strains used in this study

Plasmids	Properties	Copy number	Source
pDG30	pSC101 origin, Amp^R^, pT7: AtoB, ERG13 and tHMG1	1	Guo et al. 2018
pDG31	pBBRMCS1 origin, Chl^R^, pT7: IdI, ERG8, MVD1 and ERG12	10–20	Guo et al. 2018
pSY09	pSC101 origin, Amp^R^, pT7: Mva_SA110G_ and MvaE	1	This study
pSY12	pBR322 origin, Kan^R^, pT7: IspA and CS	100–200	This study
pSY13	pBR322 origin, Kan^R^, pT7: IspA, CS and IdI	100–200	This study
Strains			
SY01	BL21(DE3) derivative: pSY09 carrying Mva_SA110G_ and MvaE, pDG31 carrying IdI, ERG8, MVD1 and ERG12, and pSY12 carrying IspA and CS		This study
SY02	BL21(DE3) derivative: pDG30 carrying AtoB, ERG13 and tHMG1, pDG31 carrying IdI, ERG8, MVD1 and ERG12, and pSY12 carrying IspA and CS		This study
SY03	BL21(DE3) derivative: pDG30 carrying AtoB, ERG13 and tHMG1, pDG31 carrying IdI, ERG8, MVD1 and ERG12, and pSY13 carrying IspA, CS and IdI		This study

### Shake-flask cultures

Precultures of *E. coli* strains with recombinant plasmid were grown in LB at 37 °C. For production experiments the cells were transferred to 100 mL of YT medium containing 20 g/L of glucose as previously described by Kong (Kong et al. 2020), cultivated at 30 °C and induced with IPTG (0.1 mM). Chloromycetin (36 mg/L), ampicillin (100 mg/L) and kanamycin (50 mg/L) were added to the medium, when needed. In a two-phase organic overlay-culture system, 10 ml of n-dodecane was added as the upper covering extraction system.

### Glucose assays

Residual glucose concentrations were measured with a Glucose Assay Kit (Cat. MAK263, Merck). A glucose standard solution was used to create a calibration curve. Fermentation broth samples were thawed and centrifuged at 6000 *g* for 5 min to isolate supernatants for glucose content analysis.

### GC/MS analysis of τ-cadinol

Cells from 5 mL culture broth were lysed with glass beads (0.1 mm) by vigorous vortexing for 5 min. Equal volume of ethyl acetate is used to extract τ-cadinol. In a two-phase organic overlay-culture system, the n-dodecane phase was diluted in ethyl acetate with 10:1 prior to GC/MS analysis. The ethyl acetate phase was analyzed using an Agilent 7890A GC/MS equipped with a HP–5MS column. After splitless injection, the initial temperature of 80 °C was increased for 20 °C/min to 260 °C, maintain 260 °C for 8 min. Methyl pentadecanoate was used as internal standard for quantitative τ-cadinol. Compound identification was performed using NIST and Adams mass spectra databases.

## Results

### Construction of τ-cadinol biosynthetic pathway in *E. coli*

**Fig. 2:**
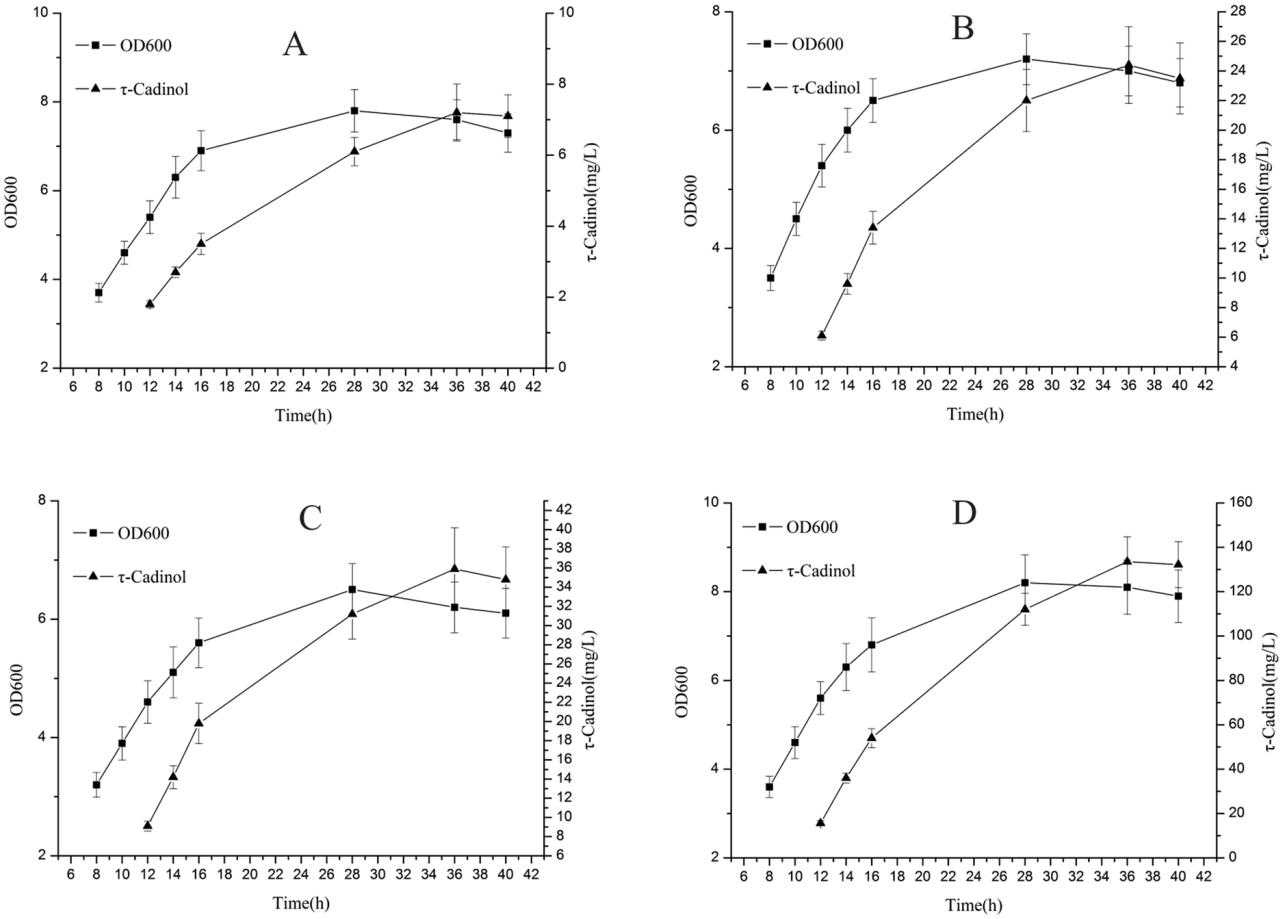
The time course of cell OD600 and τ-cadinol concentration of engineered *E. coli* strains in shake flasks. **A** SY01 strain; **B** SY02 strain; **C** SY03 strain; **D** SY03 strain with overlay-solvent

Dimethylallyl diphosphate (DMAPP) and isopentenyl diphosphate (IPP) are two precursor units of terpenoids. *E. coli* synthesizes DMAPP and IPP through the MEP pathway. However, due to the regulatory mechanism in *E. coli*, the production of target products by the MEP pathway is restricted (Martin et al. 2003). To get rid of regulatory constraints, we design a heterologous hybrid MVA pathway to produce τ-cadinol in *E. coli*. The heterologous hybrid MVA pathway is mainly composed of nine genes: mutated HMG-CoA synthase MvaS_A110G_ and bifunctional acetyltransferase/HMG-CoA reductase MvaE from *E. faecalis* for the conversion of acetoacetyl-CoA to mevalonate. IPP isomerase IdI and farnesyl diphosphate synthase IspA from *E. coli*, phosphomevalonate kinase ERG8, mevalonate pyrophosphate decarboxylase MVD1, mevalonate kinase ERG12 from *Saccharomyces cerevisiae* for the conversion of mevalonate to farnesyl diphosphate, τ-cadinol synthase CS from *L. angustifolia* for the conversion of farnesyl diphosphate to τ-cadinol. Plasmid pSY09 with co-expression of mvaS_A110G_ and mvaE, pDG31 with co-expression of IdI, ERG8, MVD1 and ERG12 (Guo et al. 2018), and pSY12 with co-expression of IspA and CS were introduced into BL21 (DE3), generating strain SY01. Strain with empty plasmid without the gene of interest is used as a negative control. Strain SY01 was cultured in YT medium with 20 g/L glucose. When the OD600 reaches about 0.8, IPTG at a final concentration of 0.1 mM was added to induce gene expression. τ-Cadinol was extracted from recombinant *E. coli* SY01 and analyzed by GC–MS (Additional file 1: Fig. S1). The biosynthesis of τ-cadinol was confirmed through comparison with the standard mass fractionation in GC–MS database (Additional file 1: Fig. S2). The time course of cell OD600 and τ-cadinol concentration of recombinant *E. coli* in shake flasks are shown in Fig. 2. τ-Cadinol can reach 7.2 ± 0.8 mg/L after 36 h of shake-flask fermentation (Table 3). The results indicate that the designed τ-cadinol biosynthetic pathway from glucose was effective in *E. coli*.

**Table 3:** τ-Cadinol production in engineered *E. coli* strains in shake flasks for 36 h

Strains	τ-Cadinol (mg/L)	Yield (mg/g)
SY01	7.2 ± 0.8	0.45 ± 0.05
SY02	24.4 ± 2.6	1.51 ± 0.16
SY03	35.9 ± 4.3	2.20 ± 0.26
SY03 with overlay-solvent	133.5 ± 11.2	8.04 ± 0.67

Yield calculated by milligrams τ-cadinol produced divided by grams glucose consumed

All experiments were performed in triplicate and SD is indicated

### Improving τ-cadinol biosynthesis by replacing mvaS_A110G_ and mvaE with AtoB, ERG13 and tHMG1

Mevalonate is a precursor substrate for the biosynthesis of τ-cadinol. Therefore, we hypothesized that increasing mevalonate availability would improve τ-cadinol production. For biosynthesis of mevalonate from acetyl-CoA, an earlier study showed that a hybrid pathway consists of *E. coli* acetoacetyl-CoA thiolase AtoB, *S. cerevisiae* 3-hydroxy-3-methylglutaryl-CoA synthase ERG13 and truncated HMG-CoA reductase tHMGR can enhance the metabolic flux of mevalonate in *E. coli* (Zhu et al. 2014*)*. Therefore, we evaluated the effect of replacing MvaS_A110G_ and MvaE with AtoB, ERG13 and tHMG1 on τ-cadinol synthesis. Plasmid pDG30 with co-expression of AtoB, ERG13 and tHMG1 (Guo et al. 2018), pDG31and pSY12 were introduced into BL21 (DE3), generating strain SY02. The strain SY02 produced up to 24.4 ± 2.6 mg/L τ-cadinol in shake flasks for 36 h (Table 3), which indicated that the replacement of MvaS_A110G_ and MvaE with AtoB, ERG13 and tHMG1 can effectively improve τ-cadinol production.

### Overexpression of IdI gene promotes the production of τ-cadinol

The farnesyl diphosphate synthase IspA catalyzes the formation of farnesyl diphosphate through the head-to-tail condensation of one DMAPP and two IPP (Fujisaki et al. 1990). The IPP isomerase IdI catalyzes the isomerization between DMAPP and IPP. Previous reports indicate that IdI is a rate-limiting enzyme and its overexpression can improve the biosynthesis of farnesyl diphosphate (Guo et al. 2018). Farnesyl diphosphate is the precursor substrate for the biosynthesis of τ-cadinol. Thus, it is expected to increase the production of τ-cadinol by enhancing the synthesis ability of farnesyl diphosphate. In this study, IdI was placed on the high-copy plasmid pSY12 and overexpressed together with CS and IspA. The resulting *E. coli* strain SY03 produced up to 35.9 ± 4.3 mg/L τ-cadinol in shake flasks for 36 h (Table 3), which indicated that the overexpression of IdI can effectively improve τ-cadinol production.

### Establishment of a two-phase fermentation system

**Fig. 3:**
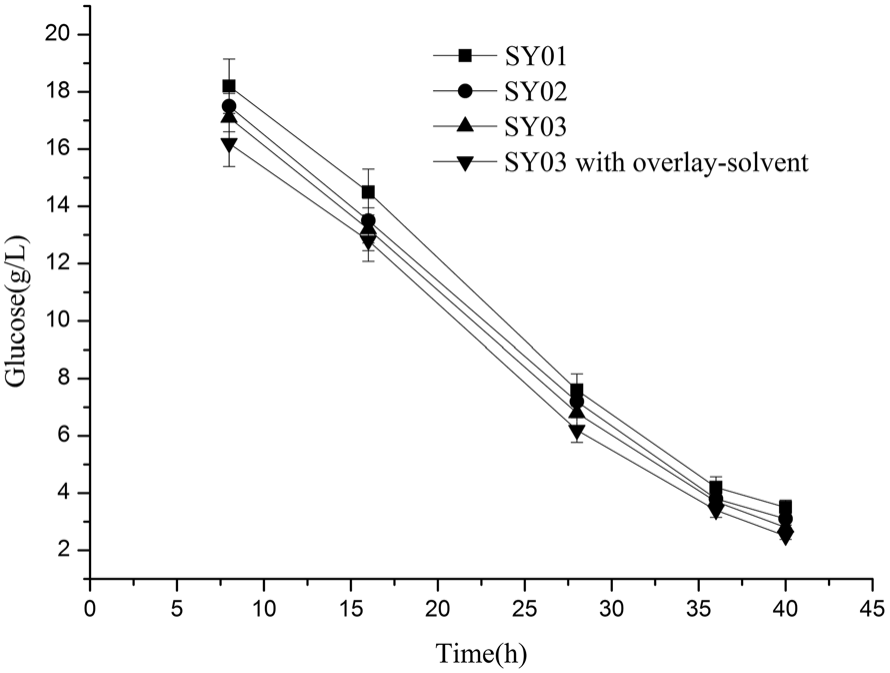
The time course of glucose concentration of engineered *E. coli* strains in shake flasks

The efficient production of terpenoids by microorganisms may be limited by end product feedback inhibition. Two-phase organic overlay-culture system has been shown to work with a large number of different recombinant strains and to improve the production capacity of the host strain by relieving the end product feedback inhibition (Li et al. 2019; Sun et al. 2021). Dodecane was considered as an ideal overlay-solvent (Frohwitter et al. 2014; Nadja et al. 2018). In this study, we have established a two-phase organic overlay-culture system with n-dodecane overlay. After 36 h of shake-flask fermentation, the τ-cadinol titer of *E. coli* strain SY03 obtained by two-phase organic overlay-culture system reached 133.5 ± 11.2 mg/L (Table 3), representing 3.7-fold improvements, when compared with that achieved in no-solvent cultures. The time course of glucose concentration of recombinant *E. coli* in shake flasks are shown in Fig. 3. τ-Cadinol yield was calculated to be 8.04 mg/g glucose and 2.9% of the theoretical yield (Table 3).

## Discussion

Terpenoids, including monoterpenes (C10), sesquiterpenes (C15), diterpenes (C20) and triterpenoids (C30), are synthesized by terpene synthases with IPP and DMAPP as substrates (Yang et al. 2013; Zhang et al. 2013; Zong et al. 2019). Traditionally, terpenoids are synthesized by plants in low amounts. The production of terpenoids by physical extraction is a time-consuming and laborious process. In addition, the long growing seasons of such plants is subject to high levels of variation due to differences in soil, climate and geography, which creates difficulties for the company's supply chain. Synthetic biology applies genetic engineering tools to build synthetic pathways in microbial cell factories to overproduce chemicals with high titers and yields. Thus, it is of great significance to develop microbial cell factories for achieving terpenoids biosynthesis. Sesquiterpene synthases catalyze the conversion from farnesyl diphosphate to a large variety of sesquiterpenes. To date, various sesquiterpenes were successfully produced by recombinant microorganism, such as β-copaene (Mischko et al. 2018), nerolidol (Qu et al. 2019), farnesol (Chonglong et al. 2010), patchoulol (Liu et al. 2021), humulene (Harada et al. 2009) and other important sesquiterpenes. Yang et al. reported that the engineered *E. coli* synthesized 220 ± 6 mg/L of β-caryophyllene from glucose by co-expression of geranyl diphosphate synthase, glucose-6-phosphate dehydrogenase and β-caryophyllene synthase genes (Du et al. 2016). Liu et al. reported that patchoulol titer in the recombinant *S. cerevisiae* reached 141.5 mg/L in a shake flask (Liu et al. 2021). Han et al. reported that engineered *E. coli* synthesized 80 mg/L of (−)-α-bisabolol in the shake-flask culture by combinatorial expression of the exogenous MVA pathway, farnesyl diphosphate synthase and (−)-α-bisabolol synthase (Han et al. 2016).

τ-Cadinol is a sesquiterpene that is widely used in perfume, fine chemicals and medicines industry. In this study, we constructed a biosynthetic pathway from glucose to τ-cadinol in *E. coli* by combinatorial expression of the exogenous MVA pathway, farnesyl diphosphate synthase IspA and τ-cadinol synthase CS. The biosynthesis of τ-cadinol was further improved by optimizing biosynthetic pathway and overexpression of rate-limiting enzyme IdI. Finally, we increased the production of τ-cadinol to 133.5 ± 11.2 mg/L by relieving end product feedback repression with a two-phase organic overlay-culture system. Zhu et al. established an in vitro reconstituted terpenoid pathway system that allows monitoring of the steady-state kinetic and biochemical parameters and the accumulation of intermediates (Zhu et al. 2014). The information thus gained could be used to guide the optimization of each biosynthetic component in *E. coli* for improvement of the production of terpenoid-derived compounds in vivo. Based on this information, we placed AtoB, ERG13 and tHMG1 genes on low-copy plasmids, IdI, ERG8, MVD1 and ERG12 genes on medium-copy plasmids, and IspA and CS genes on high-copy plasmids.

Recruiting better performing enzymes is often a good strategy to break bottlenecks in engineered MVA pathways. *S. cerevisiae* ERG12 is inhibited by substrate MVA. Therefore, the use of feedback-resistant ERG12 can reduce MVA accumulation and promote the biosynthesis of terpenoids. Chen et al. reported that lycopene titer was increased 2.4 folds by replacing the wild-type ERG12 from *S. cerevisiae* with the feedback-resistant ERG12 (Chen et al. 2018). Rad et al. reported that use of *B. licheniformis* IdI in place of *E. coli* IdI increased lycopene production 1.4 folds in engineered *E. coli* (Rad et al. 2012). HMGR consumes 2 molecules of NADPH to reduce HMG-CoA to mevalonate. Therefore, increasing the supply of NADPH will be a potentially important strategy to improve the yield of terpenoids. Recently, assembling multienzyme complexes to prevent intermediate diffusion through RIAD–RIDD interaction so as to link the upstream MVA pathway with the downstream carotenogenic pathway increased carotenoid production by 5.7-fold (Kang et al. 2019). Therefore, future efforts (for example, reduce the accumulation of intermediates by the optimization of each biosynthetic component and identification of feedback-resistant enzyme, increasing the supply of redox cofactors, discovery of novel τ-cadinol synthase, and prevent the diffusion of intermediate by assembling multienzyme complexes) will be needed to improve the production of τ-cadinol.

## Conclusions

τ-Cadinol is present in minute quantities in plants, so physical extracts of τ-cadinol is limited by the low yields. Production of τ-cadinol by microbial cell factories may be an alternative and promising approach. This study shows an efficient method for the biosynthesis of τ-cadinol in *E. coli* with the heterologous hybrid MVA pathway.

### Supplementary Information

**Additional file 1:** **Figure S1. **GC/MS analyses of τ-cadinol in engineered *E. coli* strains. Identified substances: 1, τ-cadinol; 2, methyl pentadecanoate (internal standard). **Figure S2. **Identification of τ-cadinol by Mass fractionation comparison. Mass fractionation of peaks identified in GC–MS (peak 1, RT = 7.7 min) of samples from engineered E. coli strains (red) match that obtained from τ-cadinol standard with database searches (blue). **Figure S3. **The schematic diagram of the two-phase organic overlay-culture system. **Figure S4.** Diagram of plasmids pSY 09. **Figure S5.** Diagram of plasmid pSY 13.

## Data Availability

All data generated or analyzed in this study are included in this published article and its Additional file 1.
