# Supplementary material for: De novo biosynthesis of τ-cadinol in engineered Escherichia coli
Source: Bioresour Bioprocess. 2022 Mar 21;9(1):29. doi: 10.1186/s40643-022-00521-7 (PMC10991332; doi:10.1186/s40643-022-00521-7)
Supplement: Supplementary file 1 — Additional file 1: Figure S1. GC/MS analyses of τ-cadinol in engineered E. coli strains. Identified substances: 1, τ-cadinol; 2, methyl pentadecanoate (internal standard). Figure S2. Identification of τ-cadinol by Mass fractionation comparison. Mass fractionation of peaks identified in GC–MS (peak 1, RT = 7.7 min) of samples from engineered E. coli strains (red) match that obtained from τ-cadinol standard with database searches (blue). Figure S3. The schematic diagram of the two-phase organic overlay-culture system. Figure S4. Diagram of plasmids pSY 09. Figure S5. Diagram of plasmid pSY 13. [file 40643_2022_521_MOESM1_ESM.docx]

**De novo Biosynthesis ofτ-Cadinol in Engineered *Escherichia coli***

**Yue Sun^1,2^, Shaoting Wu^2^, Xiao Fu^2^, Chongde Lai^1^*, Daoyi Guo^2^***

Corresponding author: Tel: +86-797-8393536; E-mail address: [ggdy3478@163. com](mailto:ggdy3478@163.%20com), and chongdelai_jxau@163.com

1. College of Bioscience and Bioengineering, Jiangxi Agricultural University, Nanchang 330045, China

2. Key Laboratory of Organo-Pharmaceutical Chemistry, Jiangxi Province, Gannan Normal University, Ganzhou 341000, China


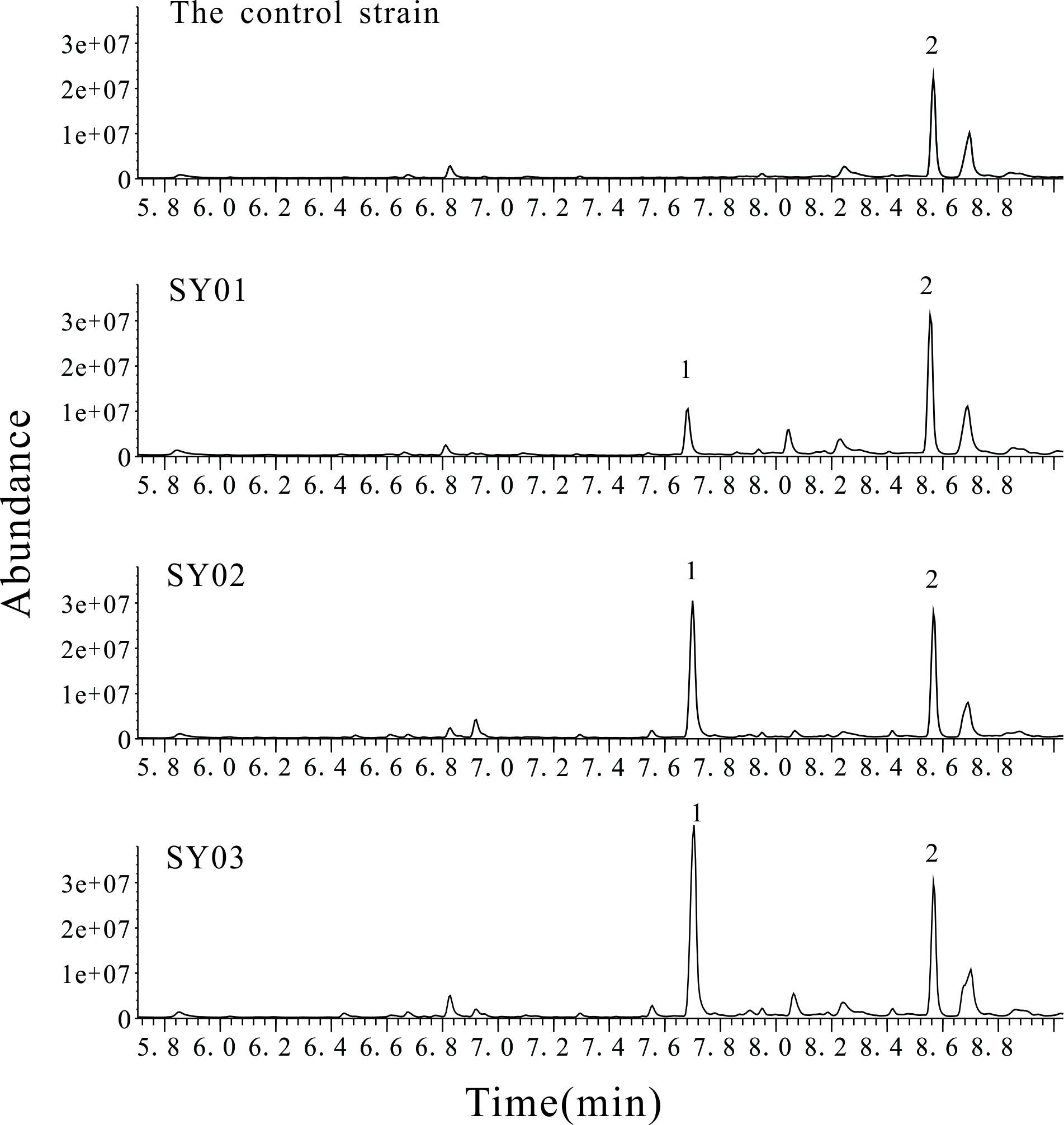


**Figure S1.** GC/MS analyses of τ-cadinol in engineered *E. coli* strains. Identified substances: 1, τ-cadinol; 2, methyl pentadecanoate (internal standard).

**
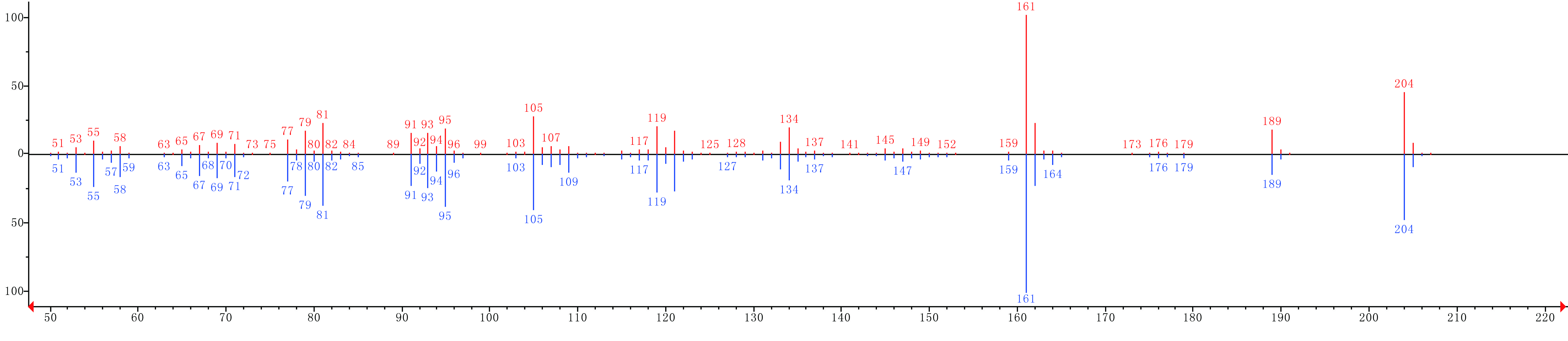
**

**Figure S2.** Identification of τ-cadinol by Mass fractionation comparison. Mass fractionation of peaks identified in GC-MS (peak 1, RT = 7.7 min) of samples from engineered E. coli strains (red) match that obtained fromτ-cadinol standard with database searches (blue).

**
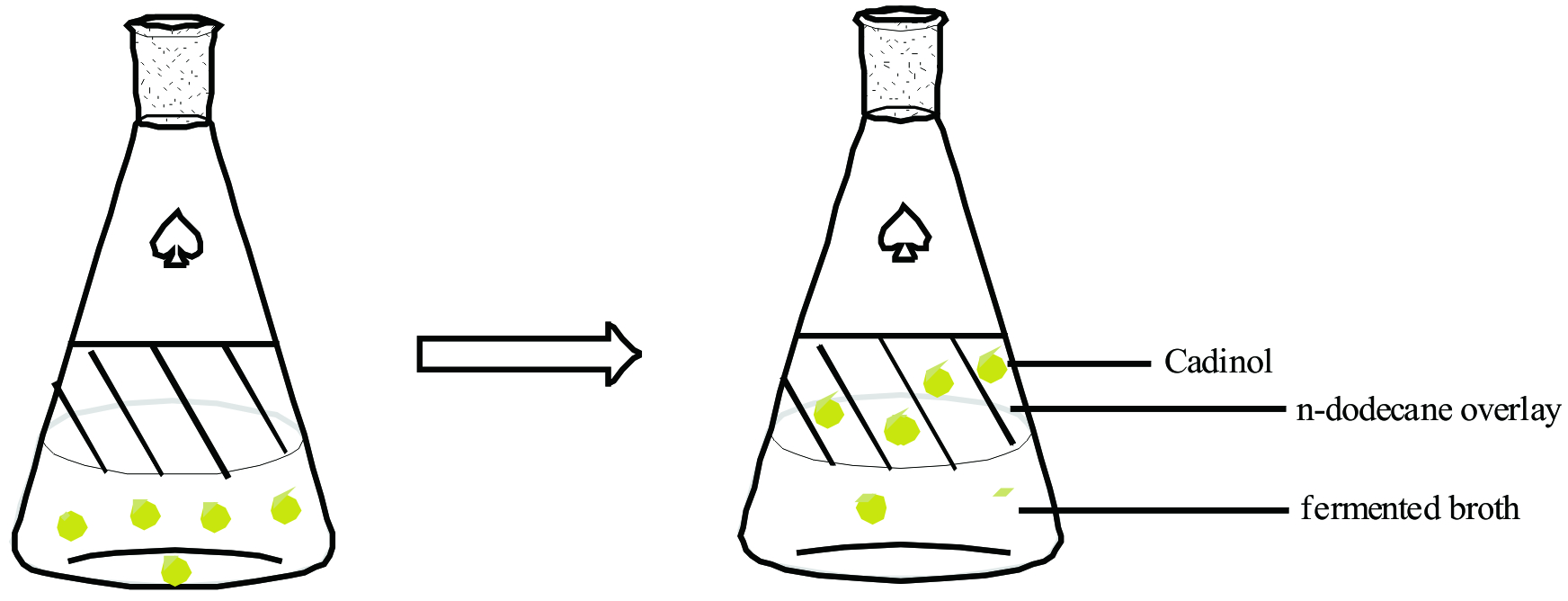
**

**Figure S3.** The schematic diagram of the two-phase organic overlay-culture system

**
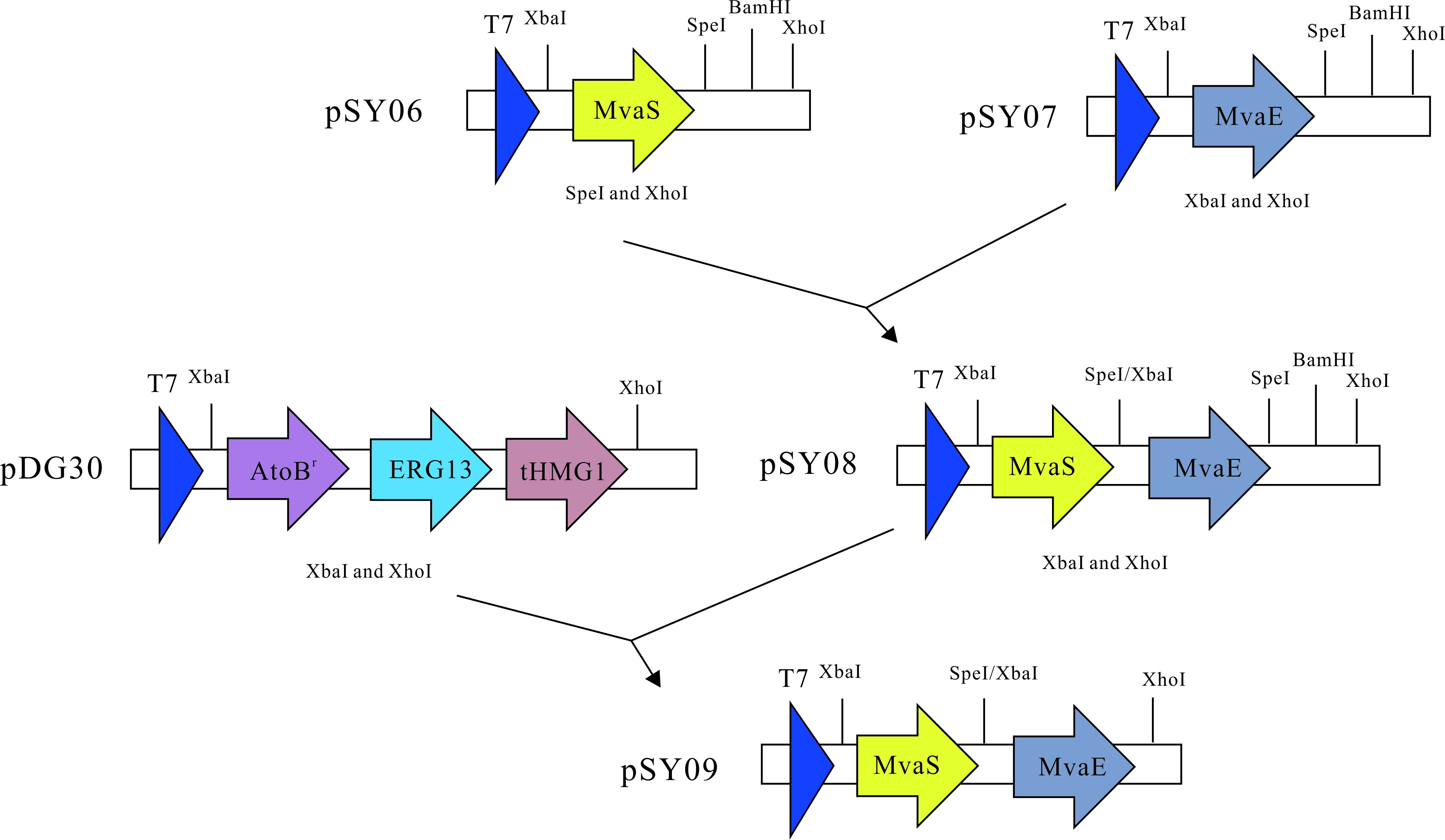
**

**Figure S4. Diagram of plasmids pSY 09**

**
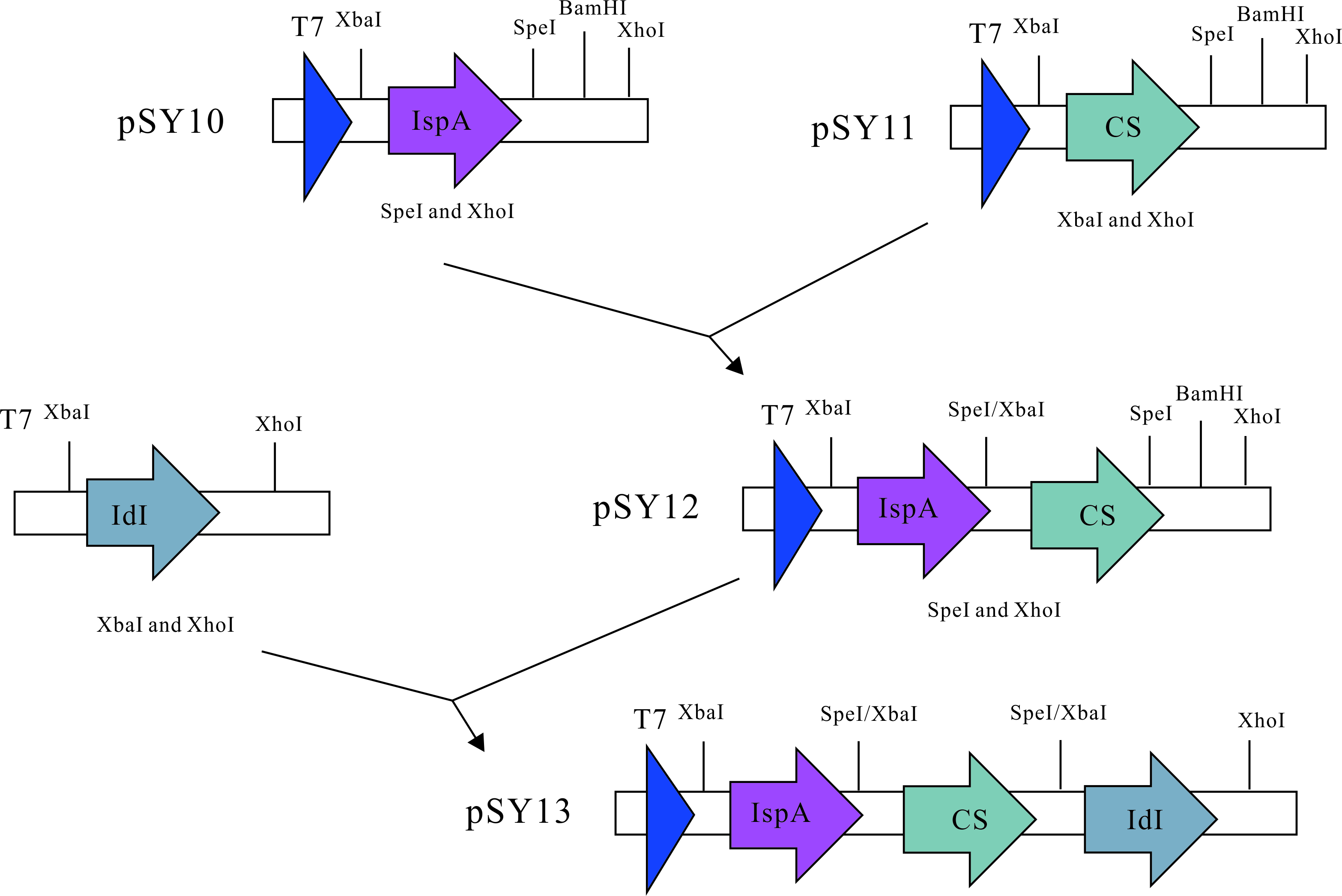
**

**Figure S5. Diagram of plasmid pSY 13**

**Syntheticτ-cadinol synthase gene**

ATGGCGACGAGCGCGGTGGTGAACTGCCTGGGCGGCGTGCGCCCGCATACCATTCGCTATGAACCGAACATGTGGACCCATACCTTTAGCAACTTTAGCATTGATGAACAAGTGCAAGGCGAATATGCGGAAGAAATTGAAGCGCTGAAACAAGAAGTGCGCAGCATGCTGACCGCGGCGACCACCTGCAAAGAACAGCTGATTCTGATTGATACCCTGGAACGCCTGGGCCTGAGCTATCATTTTGAAACCGAAATTGAACAGAAACTGAAAGAAATTATTCTGCATATTAACCGCGAAGAAGATGCGAGCGGCGGCGATTGCGATCTGTATACCACGAGCCTGGGCTTTCGCGTGATTCGTCAGCATCAGTATCATATTAGCTGCGGCGTGTTTGAAAAATATCTGGATAAAGATGGCAAATTTGAAGAAAGCCTGAGCAGCGATACCGAAGGCATTCTGAGCCTGTATGAAGCGGCGCATGTGCGCTTTCGCGATGAAACCCTGCTGCAAGAAGCGGCGCGCTTTAGCCGCCATCATCTGAAAGGCATGGAAGAAGTGCTGGAAAGCCCGCTGCGCGAAAAAGTGCAGCGCGCGCTGCAGCATCCGCTGCATCGCGATATTCCGATTTTTTATGCGCATTTTTTTATTAGCAACATTTATCAGAAAGATGATAGCCGCAACGAACTGTTACTGAAACTGGCGAAAAGCAACTTTATGTTTCTGCAGAACCTGTATAAAGAAGAACTGAGTCAGCTGAGCCGCTGGTGGAACAAATTTGATCTGAAAAGCAAACTGCCGTATGCGCGCGATCGCCTGGTGGAAGCGTATATTTGGGGCGTGGGCTATCATTATGAACCGCGCTATGCGTATGTGCGCCGCGGCCTGGTGATTGGCATTCAGATTATTGCGATTATGGATGATACCTATGATAACTATGCGACCGTGGATGAAGCGCAGCTGTTTACCGAAATGTTTGAACGCTGGAGCATGGATGGCATTGATGGCGTGCCGGATTATCTGAAAATTGCGTATCATTTTGTGGTGAGCGCGTTTGAAGATTATGAACGCGATGCGGGCAAACTGGGCAAACAGTTTGCGGCGCCGTATTTTAAACAGACCATTCAGCAGCTGGCGCGCGCGTATAACCAAGAACTGAAATGGGTGATGGGCACGCAGAGCATGCCGAGCTTTCAAGATTATGCGAAAAACAGCGAAATTACGAGCTGCATTTATATTATGAGCGCGAGCGTGTTTCATGGCCTGGAAAGCGTGACCCAAGAAACCATTGATTGGCTGAAAAACGAACCGAACTTTGCGGTGAGCACCGGCATGATTGGCCGCTATTGGGATGATATTGGCAGCCATGAACGCGAAAGCCGCGGCGGCAAAATGCTGACCGCGGTGGGTTGCTATATGAAACAGTATGGCGTGAGCAAAAAAGAAGCGGTGCGCAAATTTCGCGAACAAGTGGAAGATCTGTGGAAAGATGTGAACAAAGGCTATACCGCGATGACCTGCATGCCGCGCGAAACCGCGGTGCTGTTTCTGAACTATGCGCGCATGTGCGATGCGAGCTATACCGAAAACAACGATGATGGCTATACCGATCCGGATTTTAGCAAACGCAAAATTAGCGCGCTGTTTTTAGATCCGCTGGTGTTTTAA

**Synthetic MvaS_A110G_ gene**

ATGACCATTGGTATTGATAAAATCAGCTTTTTTGTGCCGCCGTATTACATTGACATGACCGCACTGGCCGAGGCACGCAATGTTGATCCTGGCAAATTCCATATTGGCATCGGCCAGGATCAGATGGCAGTGAACCCGATCAGCCAGGACATTGTGACCTTTGCCGCAAACGCAGCCGAAGCCATCCTGACCAAAGAGGACAAAGAAGCTATCGACATGGTGATCGTTGGCACCGAGAGCAGCATTGACGAGAGTAAAGCCGCAGCAGTGGTTTTACACCGTCTGATGGGCATCCAACCGTTTGCCCGCAGCTTCGAGATTAAAGAAGGTTGTTATGGTGCCACCGCCGGCCTGCAGCTGGCAAAAAACCACGTTGCACTGCATCCGGACAAGAAAGTTCTGGTGGTTGCCGCCGACATCGCAAAATATGGCCTGAACAGCGGCGGTGAACCTACCCAAGGTGCCGGTGCAGTGGCAATGCTGGTGGCCAGCGAACCGCGCATTCTGGCCCTGAAAGAAGACAACGTGATGCTGACCCAGGACATCTATGACTTTTGGCGTCCGACCGGCCATCCGTATCCGATGGTGGATGGCCCGCTGAGCAATGAAACCTACATTCAGAGCTTCGCCCAGGTGTGGGATGAGCACAAAAAACGTACCGGCCTGGACTTTGCCGATTATGATGCCCTGGCCTTCCACATTCCGTACACCAAAATGGGCAAGAAGGCCCTGCTGGCCAAAATCAGCGACCAAACCGAGGCAGAACAGGAACGTATTCTGGCCCGTTACGAGGAGAGCATCATCTATAGTCGCCGCGTGGGCAATCTGTACACCGGCAGCTTATATCTGGGCCTGATCAGCCTGCTGGAAAACGCCACCACCCTGACCGCCGGCAACCAGATTGGTCTGTTTAGCTATGGCAGCGGCGCAGTGGCAGAATTTTTCACAGGCGAACTGGTGGCAGGCTACCAGAACCATCTGCAGAAAGAGACCCATCTGGCCCTGCTGGATAATCGCACCGAACTGAGCATCGCCGAATATGAAGCCATGTTCGCCGAAACCCTGGATACCGACATCGATCAGACACTGGAGGACGAGCTGAAATACAGCATCAGCGCCATTAATAACACCGTGCGCAGCTATCGCAACTAA

**Synthetic MvaE** **gene**

ATGAAGACCGTTGTGATTATTGACGCACTGCGCACCCCGATCGGTAAATATAAGGGTAGCCTGAGCCAGGTTAGCGCCGTTGATTTAGGTACCCATGTGACCACCCAGCTGCTGAAACGTCACAGCACCATCAGTGAAGAAATTGATCAGGTTATCTTTGGCAACGTGCTGCAAGCAGGCAATGGCCAAAACCCTGCCCGCCAAATTGCCATTAACAGTGGCCTGAGCCATGAGATTCCGGCAATGACCGTGAACGAGGTGTGCGGCAGTGGTATGAAAGCCGTGATCCTGGCCAAGCAATTAATCCAACTGGGCGAGGCCGAAGTGCTGATTGCCGGCGGCATTGAGAATATGAGCCAGGCCCCGAAATTACAGCGTTTCAATTACGAGACCGAAAGCTACGACGCCCCGTTCAGTAGCATGATGTATGACGGCCTGACCGATGCATTTAGTGGTCAAGCCATGGGCCTGACCGCCGAAAACGTGGCCGAAAAGTATCACGTTACCCGCGAAGAACAGGACCAGTTCAGCGTTCATAGCCAGCTGAAGGCCGCCCAGGCACAAGCCGAGGGCATTTTTGCCGATGAAATCGCACCGCTGGAAGTTAGCGGCACACTGGTGGAGAAGGACGAAGGCATTCGTCCTAATAGCAGCGTGGAGAAGCTGGGTACACTGAAAACCGTGTTCAAAGAAGATGGTACCGTGACAGCAGGCAACGCCAGTACAATCAACGATGGCGCCAGCGCACTGATCATTGCCAGTCAGGAGTACGCAGAAGCCCACGGTCTGCCTTATCTGGCCATTATTCGCGACAGTGTGGAAGTGGGTATCGATCCGGCCTACATGGGTATCAGTCCGATTAAAGCAATTCAGAAGCTGCTGGCCCGTAACCAGCTGACAACCGAAGAAATCGACCTGTACGAAATCAATGAGGCCTTCGCCGCCACCAGTATTGTGGTTCAGCGTGAACTGGCCCTGCCGGAGGAGAAAGTGAACATCTACGGCGGCGGTATTAGTCTGGGTCATGCCATTGGTGCCACAGGCGCCCGCCTGCTGACAAGTCTGAGCTATCAGCTGAATCAGAAAGAGAAAAAATACGGCGTGGCAAGCCTGTGCATTGGCGGCGGCCTGGGTCTGGCAATGTTACTGGAGCGCCCGCAGCAGAAGAAAAATAGCCGTTTTTACCAGATGAGCCCGGAAGAACGCCTGGCAAGCTTACTGAACGAAGGCCAGATTAGCGCAGATACCAAGAAAGAGTTCGAAAATACCGCACTGAGTAGTCAGATTGCAAATCATATGATTGAAAACCAGATTAGTGAGACAGAGGTGCCTATGGGCGTTGGCCTGCATCTGACCGTTGACGAGACAGATTACCTGGTGCCTATGGCAACCGAAGAACCTAGCGTGATTGCCGCACTGAGCAACGGCGCCAAAATTGCACAGGGCTTTAAGACAGTTAACCAGCAGCGCCTGATGCGCGGCCAGATTGTGTTCTATGATGTGGCCGACGCAGAAAGCCTGATTGACGAGCTGCAGGTTCGCGAGACCGAGATCTTTCAGCAGGCAGAACTGAGCTATCCGAGCATTGTGAAACGCGGCGGCGGTCTGCGTGACCTGCAGTATCGCGCCTTCGATGAAAGCTTCGTGAGCGTGGACTTTCTGGTGGACGTTAAAGACGCCATGGGTGCAAATATCGTGAATGCCATGCTGGAAGGCGTGGCAGAACTGTTCCGTGAGTGGTTTGCCGAGCAAAAAATCCTGTTCAGCATTCTGAGCAACTACGCAACAGAGAGTGTTGTGACCATGAAGACCGCAATTCCGGTTAGTCGCCTGAGCAAGGGCAGCAACGGTCGCGAGATCGCAGAGAAAATCGTGCTGGCAAGTCGCTACGCAAGCTTAGATCCTTACCGCGCCGTGACCCATAATAAAGGCATTATGAATGGTATCGAAGCAGTTGTTCTGGCCACCGGCAATGATACACGCGCAGTGAGTGCAAGCTGTCACGCCTTCGCAGTGAAAGAAGGCCGCTACCAGGGTCTGACCAGCTGGACCCTGGATGGTGAGCAGTTAATTGGCGAGATTAGCGTTCCTCTGGCCTTAGCAACAGTGGGCGGCGCAACAAAGGTGCTGCCGAAAAGTCAGGCAGCCGCCGATCTGCTGGCCGTGACAGATGCCAAAGAACTGAGCCGCGTGGTTGCCGCCGTGGGTTTAGCACAAAACCTGGCAGCCCTGCGTGCACTGGTGAGCGAAGGCATCCAGAAGGGCCATATGGCCTTACAGGCACGCAGTCTGGCCATGACCGTGGGTGCCACCGGCAAAGAAGTGGAGGCCGTGGCCCAGCAGCTGAAGCGTCAGAAGACCATGAACCAGGACCGTGCCCTGGCCATCCTGAACGACCTGCGTAAACAGTAA
